# Deep-learning-based risk stratification for mortality of patients with acute myocardial infarction

**DOI:** 10.1371/journal.pone.0224502

**Published:** 2019-10-31

**Authors:** Joon-myoung Kwon, Ki-Hyun Jeon, Hyue Mee Kim, Min Jeong Kim, Sungmin Lim, Kyung-Hee Kim, Pil Sang Song, Jinsik Park, Rak Kyeong Choi, Byung-Hee Oh

**Affiliations:** 1 Department of Emergency Medicine, Mediplex Sejong Hospital, Incheon, Korea; 2 Artificial intelligence and big data center, Sejong medical research institute, Gyeonggi, Korea; 3 Division of Cardiology, Cardiovascular Center, Mediplex Sejong Hospital, Incheon, Korea; Hospital Clinico San Carlos, SPAIN

## Abstract

**Objective:**

Conventional risk stratification models for mortality of acute myocardial infarction (AMI) have potential limitations. This study aimed to develop and validate deep-learning-based risk stratification for the mortality of patients with AMI (DAMI).

**Methods:**

The data of 22,875 AMI patients from the Korean working group of the myocardial infarction (KorMI) registry were exclusively divided into 12,152 derivation data of 36 hospitals and 10,723 validation data of 23 hospitals. The predictor variables were the initial demographic and laboratory data. The endpoints were in-hospital mortality and 12-months mortality. We compared the DAMI performance with the global registry of acute coronary event (GRACE) score, acute coronary treatment and intervention outcomes network (ACTION) score, and the thrombolysis in myocardial infarction (TIMI) score using the validation data.

**Results:**

In-hospital mortality for the study subjects was 4.4% and 6-month mortality after survival upon discharge was 2.2%. The areas under the receiver operating characteristic curves (AUCs) of the DAMI were 0.905 [95% confidence interval 0.902–0.909] and 0.870 [0.865–0.876] for the ST elevation myocardial infarction (STEMI) and non ST elevation myocardial infarction (NSTEMI) patients, respectively; these results significantly outperformed those of the GRACE (0.851 [0.846–0.856], 0.810 [0.803–0.819]), ACTION (0.852 [0.847–0.857], 0.806 [0.799–0.814] and TIMI score (0.781 [0.775–0.787], 0.593[0.585–0.603]). DAMI predicted 30.9% of patients more accurately than the GRACE score. As secondary outcome, during the 6-month follow-up, the high risk group, defined by the DAMI, has a significantly higher mortality rate than the low risk group (17.1% vs. 0.5%, p < 0.001).

**Conclusions:**

The DAMI predicted in-hospital mortality and 12-month mortality of AMI patients more accurately than the existing risk scores and other machine-learning methods.

## Introduction

In the past decades, the mortality rate of acute myocardial infarction (AMI) has improved with advances in early reperfusion therapy and adjunctive pharmacotherapy.[1] However, AMI is still the major leading cause of mortality worldwide.[2–4] Risk stratification and prognosis prediction are critical in identifying high risk patients and decision making for the treatment of patients with AMI.[5] Conventional risk scoring systems including the thrombolysis in myocardial infarction (TIMI), the global registry of acute coronary events (GRACE), and the acute coronary treatment and intervention outcomes network (ACTION) scores are widely validated and accepted tools that are estimated using patients’ clinical information.[6–8] However, these prognostic models have limitations for the current daily practice. First, these systems are questionable in contemporary practice because they had been developed 20 years ago. Additionally, as these models use only selective variables based on a conventional statistical method, there is a possibility of loss of important information.[9–12]

Recently, deep-learning has achieved high performance in several medical domains, such as image classification (e.g., detection of abnormalities in retinal funduscopic result) and clinical outcome prediction (e.g., in-hospital mortality and long-term outcomes).[13–15] An advantage of deep-learning is the automatic learning of the feature and relationship from a given data.[16] In this study, we developed and validated a deep-learning-based risk stratification for the mortality of patients with acute myocardial infarction (DAMI) using the Korean working group of myocardial infarction (KorMI) registry, a large national data.

## Methods

### Study population

We conducted a retrospective observational cohort study using data from the KorMI registry. KorMI is a prospective multicenter registry of AMI in Korean patients. All 59 cardiovascular centers in Korea were included in this study in January 2008. The full details of the KorMI registry’s aims and protocols have been published elsewhere.[17] This study was conducted in accordance with the Declaration of Helsinki and the relevant guidelines and regulations. The institutional review boards of Sejong General Hospital and Mediplex Sejong Hospital approved this study protocol and granted waivers of informed consent based on general impracticability and minimal harm. Patient information was anonymized and de-identified before the analysis. The data obtained through KorMI were the demographic information, treatment in the emergency room, laboratory results, electrocardiography findings, final diagnosis, clinical outcome during their hospital stay, and 12-month prognosis after discharge. The data were collected at each hospital using an encrypted web database.

### Data management

**Fig 1:**
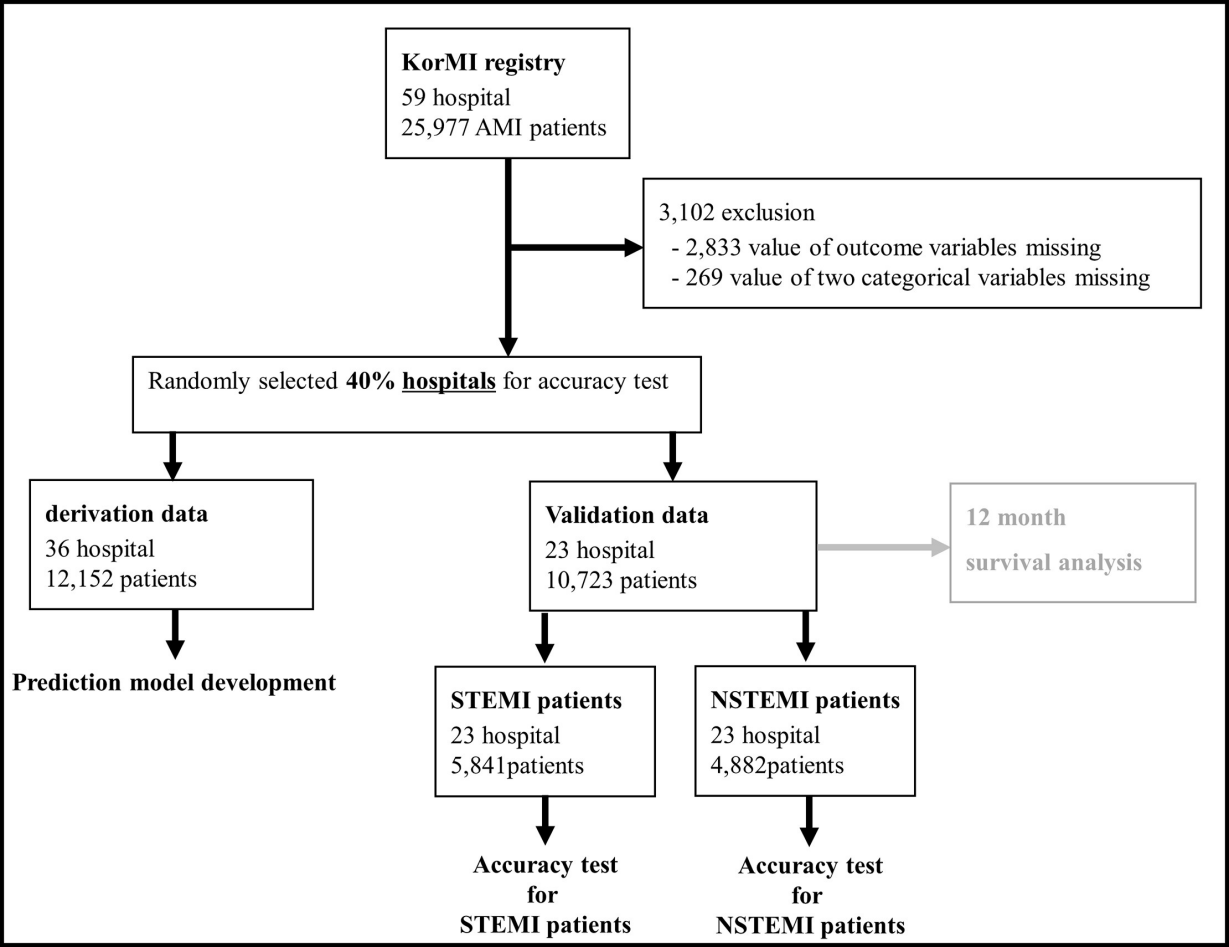
Study flow chart.

First, the study data was split according to the hospital to prepare the validation data (Fig 1). The number of hospitals contributing data to the KorMI registry was 59, and we randomly selected 60% (36 hospitals) and 40% (23 hospitals) of these hospitals from which to obtain training data and validation data, respectively. As deep-learning and machine-learning can learn the characteristics of a hospital, we divided the data into training and validation dataset not by study subjects, but by hospitals, for the purpose of confirming further exact validation tests. For more accurate validation tests and subgroup analyses, we distributed as many study subjects as possible into the validation datasets. The validation data was used to confirm whether the DAMI can be applied to other hospitals after development.

The DAMI is a risk stratification model to predict in-hospital mortality after an AMI. We used the demographic information and laboratory data of AMI patients including age, sex, body mass index, cardiac arrest before visit, systolic blood pressure, heart rate, Killip class, creatinine kinase-muscle/brain (CK-MB), glucose, C-reactive protein (CRP), creatinine, low-density lipoprotein, and elevation of the ST segment, as the predictor variables. We aim to develop the model to help physicians in deciding a treatment plan, such as performing emergent coronary angiography or mechanical circulatory support, at the time of initial evaluation and treatment. Because of this, we used the predictor variables which could be obtained at the time of initial evaluation. We used first documented values in each admission, such as a first vital heart rate at emergency department.

### Development of machine-learning prediction model

**Fig 2:**
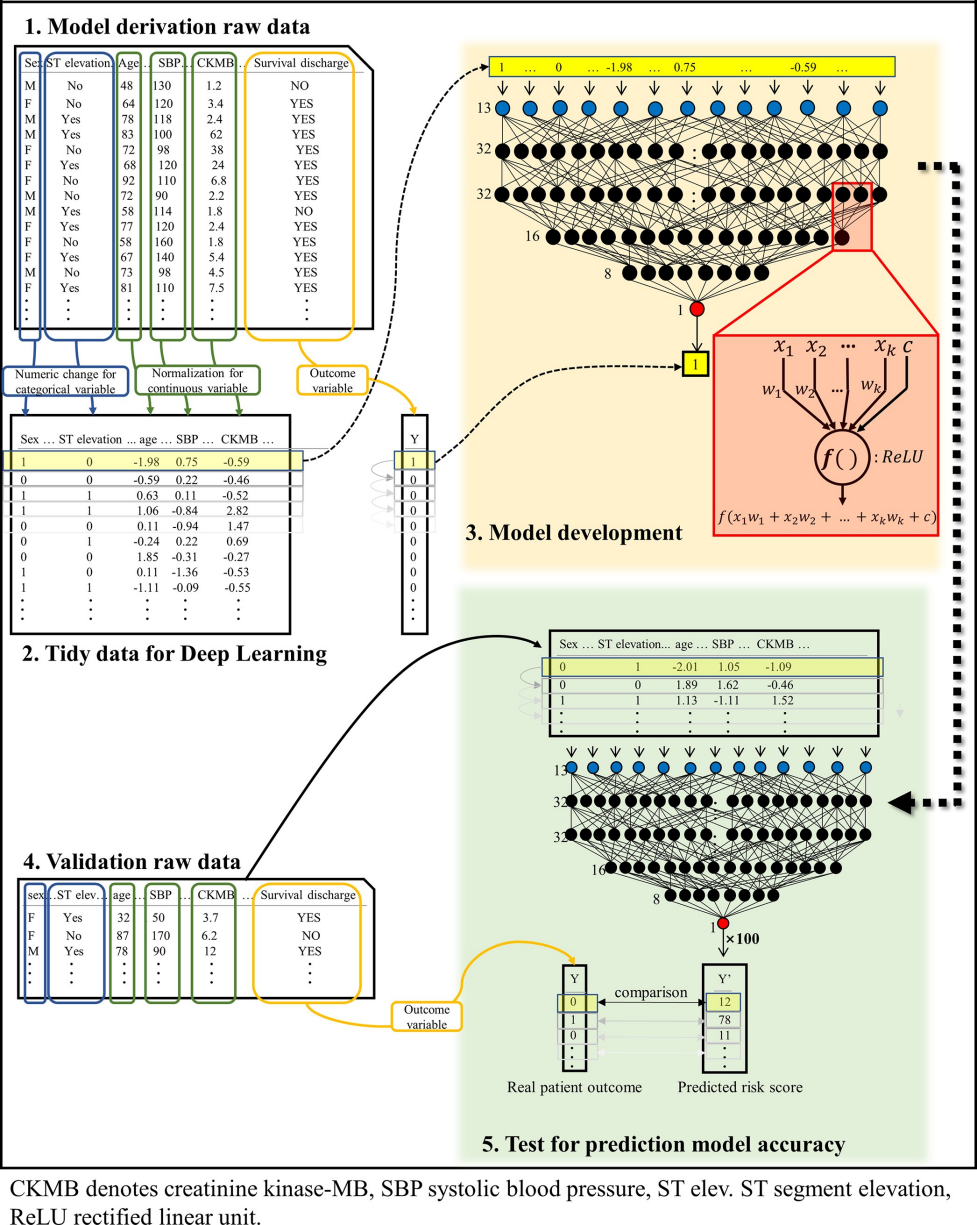
Deep-learning based model development and accuracy test. AMI denotes acute myocardial infarction, CKMB creatinine kinase-MB, CVA cerebrovascular accident, DM diabetes mellitus, HTN hypertension, PMHx past medical history, ReLU rectified linear unit.

As shown in Fig 2, we developed the DAMI using only the derivation data. The The DAMI is a multilayer perceptron (MLP) based on deep-learning, and the DAMI incorporates four hidden layers, 102 nodes, batch normalization, and dropout layers.[18–20] Because the accuracy did not increase when five or more hidden layers were added, we used four hidden layers to minimize the number of parameters to be learned. We used Tensor Flow (the Google Brain Team) as the backend.[21] Further, we used the Adagrad optimizer with the default parameters and binary-cross entropy as the loss function.[22] One node of the MLP is added by multiplying the values from the upper layer nodes (*x_k_*) by the weights (*w_k_*). The added value, (*x*_1_*w*_1_+*x*_2_*w*_2_+⋯+*x_k_w_k_*+*c*), is processed by the activation function, and the value *f*(*x*_1_*w*_1_+*x*_2_*w*_2_+⋯+*x_k_w_k_*+*c*) is sent to the next node. In this MLP, we used a rectified linear unit (ReLU) as the activation function.[23] Because maximum accuracy is observed for this predictive model using ReLU when compared with other activation functions such as soft max, linear, Tanh, leaky ReLU, and exponential linear unit, all the hyper-parameters used in the DAMI were tuned using grid search and manual tuning.

Before using the derivation data for the model development, we replaced the values of the categorical variables to numeric values and normalized the values of the continuous variables (Fig 2).[24] This data preprocessing was performed in the derivation data and validation data, separately. To train the model, we input each value of the derivation data in the input layer and adjusted the weight (*w_k_*) using the back propagation.[25] We have provided our prediction model as S1 File.

We also develop two machine-learning models: logistic regression (LR) and random forest (RF), for the performance comparison with the DAMI.[26] In the previous studies, LR and RF are the most typically used machine-learning methods and showed better performance than traditional methods in several medical domains.[27,28] The RF model consisted of 10,000 decision trees using the “randomForest” package in R (R Development Core Team, Vienna, Austria).[29,30] The LR model was derived using the “‘glmulti” packages in R.[31] We used the original Akaike IC as the information criterion and forward-backward directions for LR model selection.

### Validation of prediction model performance

After we developed the DAMI, LR, and RF models, we compared the performance of these models with the GRACE, ACTION, and TIMI scores. We compared the performance of the models using only validation data that were not used for the model development (Fig 1). We analyzed the variable importance of logistic regression, random forest, and deep-learning by using deviance difference, mean decrease Gini, and AUC difference, respectively. In the GRACE and TIMI score, the formulas for calculating the risk score differ depending on the elevation of ST segment. For this reason, we divided the validation data into ST elevation myocardial infarction (STEMI) and non-ST elevation myocardial infarction (NSTEMI) and confirmed the accuracy at each group. We used the area under the receiver operating characteristic curve (AUC) as the comparative measure. [32]

We divided the patients of the validation data into high risk, intermediate risk, and low risk groups according to the DAMI and GRACE scores. The cutoff points of GRACE score were determined in previous studies.[33] The predicted mortality of low, intermediate, high risk group of GRACE score are less than 1%, 1–3%, and over 3%, respectively. And the optimal cutoff points of DAMI score were determined when the predicted in-hospital mortality of each risk group was equal to the that of GRACE score. After dividing the risk group by the DAMI and GRACE scores, we compared the accuracy for the in-hospital mortality through the reclassification table. Further, we confirmed characteristics of the DAMI risk groups. The continuous variables were presented as the mean and standard deviation and were compared using the analysis of variance (ANOVA) test. The categorical variables are expressed as frequencies and percentages and were compared by the Chi-square statistics. We estimated the 6-month mortality rate by the DAMI risk groups using the Kaplan–Meier method.

## Results

We included 25,977 AMI patients enrolled in the KorMI registry from January 2008 to December 2013 and excluded 3,102 patients according to the criteria mentioned in Fig 1. There is observed to be no significant difference in predictor variables between included and excluded study subjects, as shown in S1 Table. The study subjects comprised 22,875 patients of 59 hospitals, where 1,081 had in-hospital mortality. In-hospital mortality for the study subjects was 4.4%. In study subjects who survived upon discharge, 6-month mortality was 2.2% and mean (± standard deviation) of time after discharge was 28.5 days (± 41.7 days). The DAMI was developed using 12,152 patients of the derivation data. The accuracy test was performed using 10,723 patients of the validation data, where STEMI and NSTEMI patients were 5,841 and 4,882, respectively (Fig 1). We provide the developed DAMI, coding book for making input tidy data, example of tidy validation data, and python code for accuracy test as a S1 File to this article.

**Fig 3:**
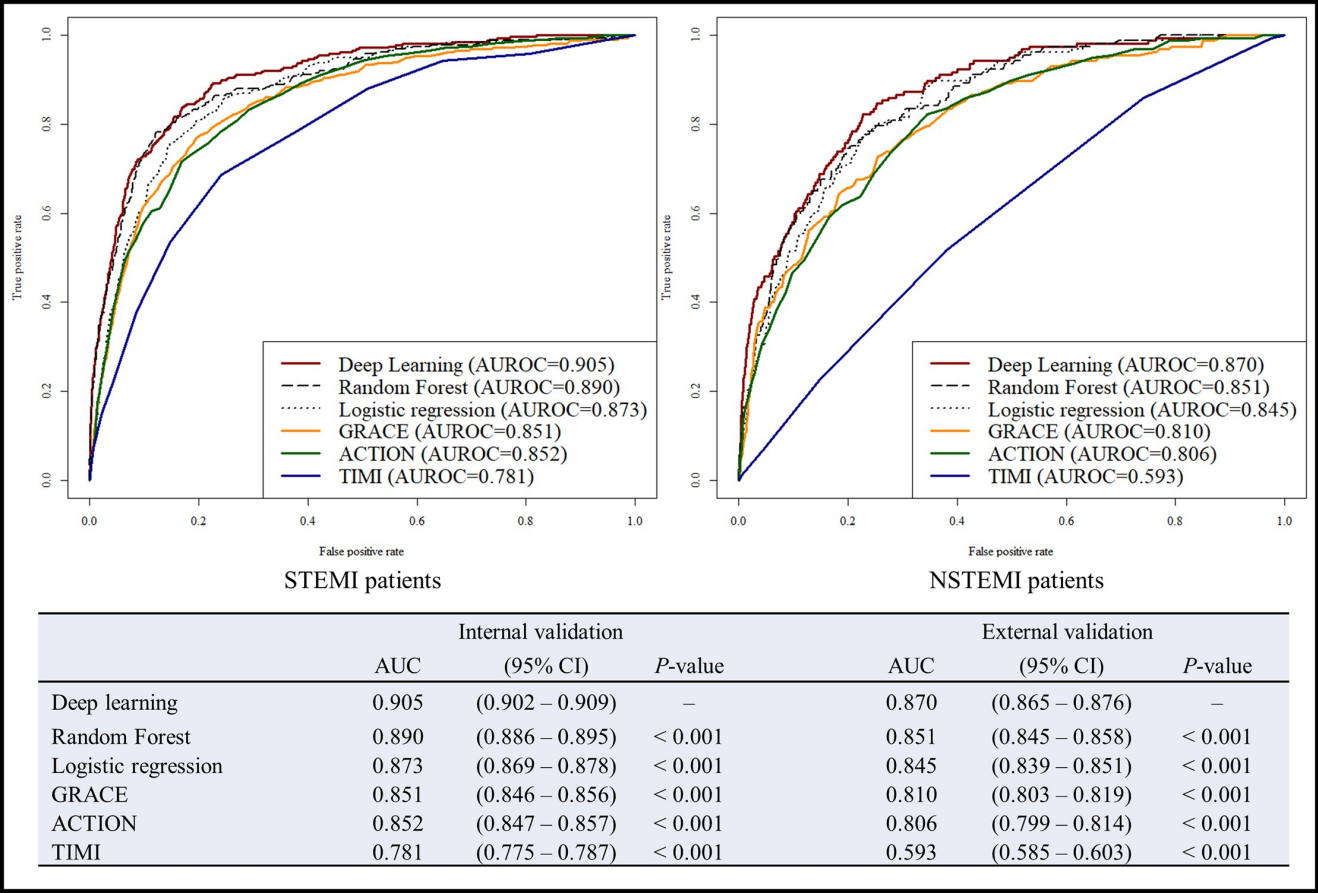
Receiver operating characteristic curve for predicting in-hospital mortality. AUC denotes area under the receiver operating characteristic curve, CI confidence interval, GRACE global registry of acute coronary event, TIMI thrombolysis in myocardial infarction.

As shown in Fig 3, during the accuracy test of STEMI patients, the AUC of the DAMI was 0.905 [95% confidence interval 0.902–0.909] and this result significantly outperformed the GRACE score (0.851 [0.846–0.856]), ACTION score (0.852 [0.847–0.857]), TIMI score (0.781 [0.775–0.787]), LR (0.873 [0.869–0.878]), and RF (0.890 [0.886–0.895]). In the NSTEMI patients group, the AUC of the DAMI was 0.870 [0.865–0.876] and this accuracy significantly outperformed the GRACE score (0.810 [0.803–0.819]), ACTION score (0.806 [0.799–0.814]), TIMI score (0.593 [0.585–0.603]), LR (0.845 [0.839–0.851]), and RF (0.851 [0.845–0.858]). The variable importance of each prediction model is shown in S2 Table. The variable importance is different for each prediction model.

**Fig 4:**
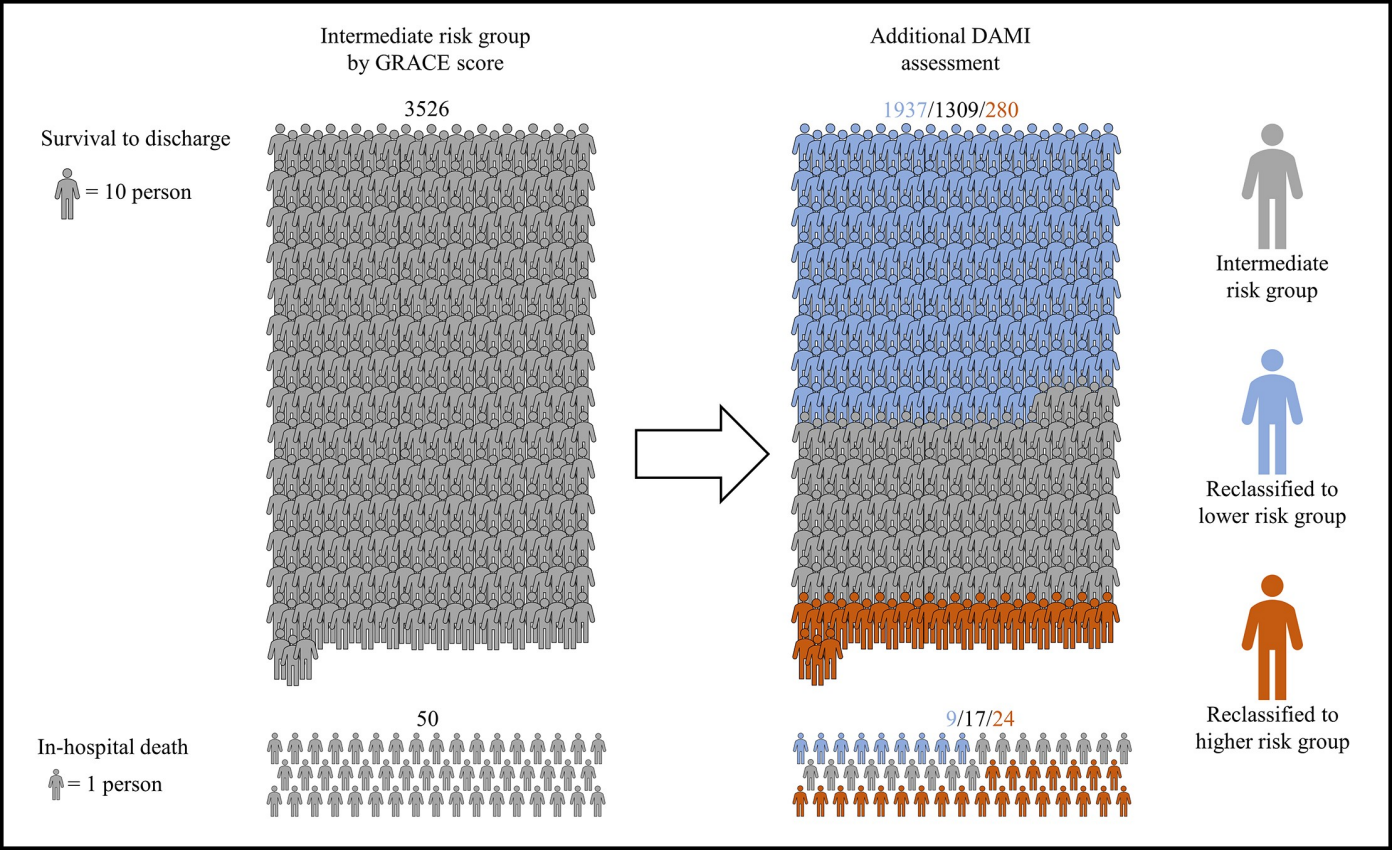
Reclassification of Individuals predicted to be at intermediate risk group by additional assessment of DAMI. DAMI denotes deep-learning-based risk stratification for the mortality of patients with AMI and GRACE denotes global registry of acute coronary event.

In the following experiments, we used the combined data from the STEMI and NSTEMI validation data. In the validation data, the cut-off scores of the DAMI risk groups were 2.3 and 7.9. With this cut-off value, the DAMI classified 2,843, 2,957, and 4,923 patients as high, intermediate, and low risk, respectively. Table 1 shows the baseline characteristics of patients between the high, intermediate, and low risk groups, defined by the DAMI. As shown in the reclassification table (Table 2), the DAMI predicted 34 in-hospital mortality patients and 3678 survival discharge patients more accurately than the GRACE score. And the DAMI predicted 31 in-hospital mortality patients and 573 survival discharge patients more incorrectly than the GRACE score. In 3526 patients who were in intermediate group by GRACE score, 1937 patients were reclassified to low risk group and in 50 patients of in-hospital death, 24 patients were reclassified to high risk group and 9 patients were reclassified to low risk group by DAMI score (Fig 4).

**Table 1:** Baseline characteristics of study subjects.

	Derivation data (n = 12,152)		Validation data		
		All population (n = 10,723)	Low risk group (n = 4923)	Intermediate risk group (n = 2957)	High risk group (n = 2843)
**Baseline characteristics**					
Age, year	64.0 ± 12.8	63.63 ± 12.6	56.1 ± 10.7	68.3 ± 9.6	71.9 ± 10.8
Female (%)	3521(29.0%)	2992(27.9%)	778(15.8%)	1033(34.9%)	1181(41.5%)
Body mass index, kg/m^2^	24.0 ± 3.6	24.0 ± 3.3	24.9 ± 3.6	23.4 ± 2.7	23.1 ± 3.1
Hypertension (%)	6218(51.2%)	5173(48.2%)	1947(39.5%)	1541(52.1%)	1685(59.3%)
Diabetes (%)	3432(28.2%)	2885(26.9%)	866(17.6%)	891(30.1%)	1128(39.7%)
Dyslipidemia (%)	1553(12.8%)	1163(10.8%)	642(13.0%)	290(9.8%)	231(8.1%)
Current smoking (%)	4921(40.5%)	4321(40.3%)	2567(52.1%)	947(32.0%)	807(28.4%)
Congestive heart failure (%)	261 (2.1%)	134 (1.2%)	6 (0.1%)	35 (1.2%)	93 (3.3%)
Chronic kidney disease (%)	221 (1.8%)	219 (2.0%)	19 (0.4%)	44 (1.5%)	156 (5.5%)
Chronic lung disease (%)	241 (2.0%)	129 (1.2%)	30 (0.6%)	36 (1.2%)	63 (2.2%)
Chronic liver disease (%)	102 (0.8%)	83 (0.8%)	37 (0.8%)	24 (0.8%)	22 (0.8%)
Cancer (%)	262 (2.2%)	143 (1.3%)	39 (0.8%)	54 (1.8%)	50 (1.8%)
Prior AMI (%)	336 (2.8%)	398 (3.7%)	141 (2.9%)	116 (3.9%)	141 (5.0%)
Prior CVA (%)	861 (7.1%)	635 (5.9%)	171 (3.5%)	191 (6.5%)	273 (9.6%)
Prior PCI (%)	858 (7.1%)	563 (5.3%)	223 (4.5%)	181 (6.1%)	159 (5.6%)
Prior CABG (%)	80 (0.7%)	87 (0.8%)	16 (0.3%)	34 (1.1%)	37 (1.3%)
Family history of heart disease (%)	1143 (9.4%)	697 (6.5%)	464 (9.4%)	127 (4.3%)	106 (3.7%)
Past medical history					
Aspirin (%)	2101 (17.3%)	1090(10.2%)	401 (8.1%)	340 (11.5%)	349 (12.3%)
Anti-platelet (%)	821 (6.8%)	467 (4.4%)	163 (3.3%)	133 (4.5%)	171 (6.0%)
Anti-coagulant (%)	70 (0.6%)	56 (0.5%)	22 (0.4%)	14 (0.5%)	20 (0.7%)
Statin (%)	1197 (9.9%)	589 (5.5%)	226 (4.6%)	176 (6.0%)	187 (6.6%)
**Initial presentation**					
Chest pain (%)	10257(84.4%)	7286(67.9%)	3593(73.0%)	2013(68.1%)	1680(59.1%)
Dyspnea (%)	3449(28.4%)	1763(16.4%)	468(9.5%)	431(14.6%)	864 (30.4%)
Killip Class					
Class I—II (%)	10687(87.9%)	9307(86.8%)	4901(99.6%)	2816(95.2%)	1590(55.9%)
Class III (%)	808 (6.6%)	877 (8.2%)	22 (0.4%)	132 (0.4%)	723 (25.4%)
Class IV (%)	657 (5.4%)	539 (5.0%)	0 (0%)	9 (0.3%)	530 (18.6%)
Systolic blood pressure, mmHg	128.8 ± 28.1	130.0 ± 26.6	136.8 ± 24.7	128.6 ± 24.7	119.7 ± 28.0
Diastolic blood pressure, mmHg	78.1 ± 16.2	79.7 ± 15.8	83.8 ± 15.0	78.5 ± 14.3	73.7 ± 16.7
Heart rate, bpm	78.9 ± 20.0	77.6 ± 18.3	73.5 ± 14.3	76.7 ± 16.0	85.6 ± 23.5
ST segment elevation (%)	6494(53.4%)	5841(54.5%)	2602(52.9%)	1616(54.6%)	1623(57.1%)
Cardiac arrest (%)	242 (2.0%)	216 (2.0%)	7 (0.1%)	20 (0.7%)	189 (6.6%)
**Initial Laboratory findings**					
Glucose, mg/dL	171.7 ± 81.3	170.1 ± 81.3	147.1 ± 52.9	169.7 ± 76.9	210.2 ±106.8
Creatinine, mg/dL	1.2 ± 1.0	1.1 ± 1.0	0.9 ± 0.3	1.1 ± 0.9	1.6 ± 1.6
CK-MB, ng/mL	121.3 ± 196.7	112.8 ±234.9	101.7 ±167.8	105.3 ±162.0	140.1 ±362.0
Troponin I, ng/mL	38.7 ± 111.1	40.9 ±98.0	34.6 ±58.4	41.0 ± 98.4	51.7 ± 141.7
Total cholesterol, mg/dL	181.7 ± 45.4	182.8 ± 44.5	192.1 ± 44.0	179.4 ± 41.8	170.3 ± 44.3
-cholesterol, mg/dL	114.4 ± 38.6	114.5 ± 38.2	122.2 ± 39.7	110.9 ± 35.9	104.7 ± 34.8

AMI indicates acute myocardial infarction; CABG, coronary artery bypass graft; CK, creatinine kinase; CVA, cerebrovascular accident; ECG, electrocardiography; LDL, low density lipoprotein; NSTEMI, non-ST-elevation myocardial infarction; PCI, percutaneous coronary intervention; and STEMI ST-elevation myocardial infarction.

**Table 2:** Reclassification table.

		Reclassified predicted risk with DAMI score			Subjects reclassified with		
		Low risk group	Intermediate risk group	High risk group	Increased risk	Decreased risk	Net correctly reclassified (%)
	**In-hospital mortality patients (N = 459)**						
**Predicted risk with GRACE**	**Low risk group**	7	5	5	34 (7.2%)	31 (6.6%)	**3 (0.6%)**
	**Intermediate risk group**	9	17	24			
	**High risk group**	0	22	384			
	**Survival discharge patients (N = 8907)**						
**Predicted risk with GRACE**	**Low risk group**	2580	253	40	573 (5.6%)	3678 (35.9%)	**3105 (30.3%)**
	**Intermediate risk group**	1937	1309	280			
	**High risk group**	390	1351	2110			

**Fig 5:**
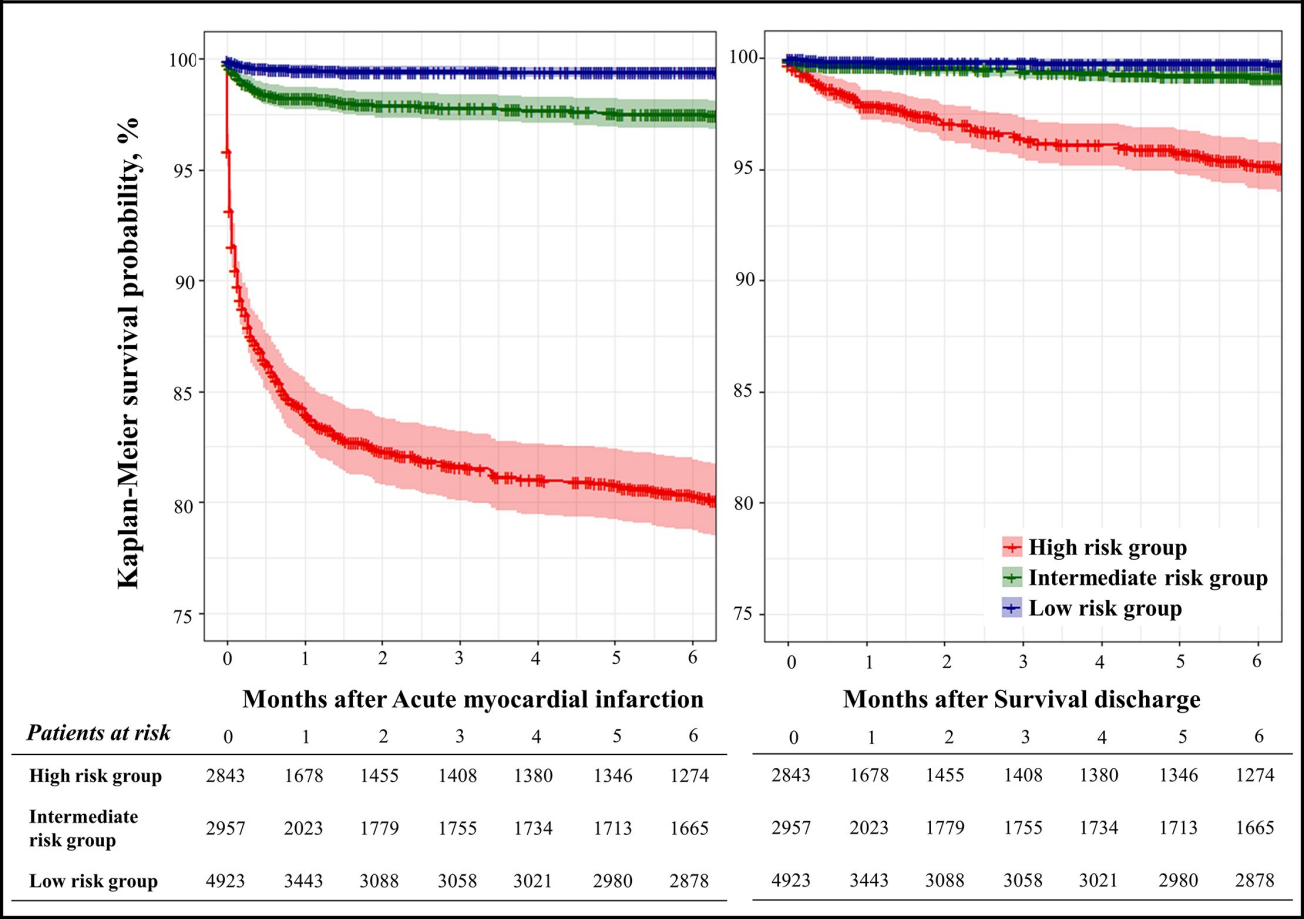
Kaplan-Meier survival curve stratified by deep-learning model risk score group.

For the analysis of mortality during the 6-month period, we considered the validation data of 10,723 patients (Fig 1). As shown in the Kaplan-Meier survival curves of Fig 5, the high-risk group defined by the DAMI shows a significantly higher hazard than the low risk group. The high-risk group, defined by the DAMI, has a significantly higher mortality rate than the low-risk group (17.1% vs. 0.5%, p < 0.001).

## Discussion

In this study, we developed a risk stratification model for the mortality of patients with AMI using deep-learning from a large prospective national registry. By the accuracy test, this study revealed that the accuracy performance of the deep-learning model was excellent for predicting the prognosis and is better than the conventional risk-prediction model. To the best of our knowledge, this study is the first to predict AMI patient outcomes using deep-learning.

The TIMI and GRACE scores are extensively validated, traditional models for risk stratification following AMI.[33,34] The previous validation studies have reported that the AUCs of the TIMI and GRACE scores were 0.60–0.70 and 0.80–0.85, respectively.[35] It is confirmed in this large study population as 0.59–0.78 for the TIMI score and 0.81–0.85 for the GRACE score, implying moderate accuracy for predicting the mortality of AMI patients.

However, several notable limitations exist in the TIMI and GRACE scores. First, these models were developed based on the AMI patients’ data between the 1990s and early 2000s. In the past decade, early reperfusion therapy and the routine usage of drug-eluting stents have become routine. The benefit of intensive statin therapy was confirmed and potent antiplatelet agents, such as prasugrel and ticagrelor were introduced in our daily practice.[36–39] In addition, these scoring systems are different for STEMI and NSTEMI. Recently, Song et al. reported a new scoring system for predicting the outcomes in survivors treated with guideline-adherent optimal therapies after AMI using the conventional statistical approach, which had better discrimination power than the GRACE model or other scoring system.[40] The DAMI risk stratification model was developed based on relatively recent data, which can better predict the mortality of AMI patients in the current practice. And the status of ST segment is included in DAMI algorithm, DAMI can equally well predict the mortality of AMI regardless of ST elevation. Next, the old models, used in TIMI, GRACE, and ACTION, inevitably restrict the numbers of predictive factors, because these models were developed by the conventional statistical approach using the logistic regression model that contains limitation including the fixed assumptions on data behavior, and the necessity to preselect variables in the development phase, thus leading to potential information loss.[9–12] Unlike the conventional statistical approach, deep-learning does not require the preselection of important variables, and the less important variables are naturally ignored in the model fitting.[41–43] Further, deep-learning does not limit the number of input predictive factors and can use all available information without potential loss. Subsequently, the old models cannot reflect the relationship between variables. This is because the risk is measured only by the sum of the variables. Meanwhile, deep-learning obtains the relationship between the variables, as shown in Fig 2, unlike conventional methods.[16]

A previous study attempted to predict a 30-day mortality after ST-elevation myocardial infarction using conventional machine-learning methods including LR and RF and confirmed that RF performed the best.[28] However, no significant difference in performance is shown between the RF and GRACE scores. In that study, the machine-learning requires a feature-selection step before developing a predictive model. The feature selection is to delete variables that are less relevant to the prediction outcome and leads to potential information loss.[44] An important advantage of the deep-learning compared with conventional machine-learning, such as LR and RF, is feature learning.[16] In our study, feature learning is applied to obtain useful features to predict the endpoint of an AMI patient. Using a large amount of data, the deep-learning model automatically learns the features and conducts the given tasks such as classification and detection. This is why deep-learning shows better results than traditional machine-learning.[41–43]

Deep-learning and machine-learning are used to obtain the relationship between the predictor variables and outcome variable, rather than creating a rule based on medical knowledge. Hence, the performance of machine-learning and deep-learning is not guaranteed in other situations as the algorithms can memorize only the characteristics of the derivation data. Wolpert explains the no-free-lunch theorem; if optimized in one situation, a model cannot produce good results in other situations.[45] Hence, we conducted an accuracy test using data which were not used for the model derivation. As deep-learning and machine-learning can learn the characteristics of a hospital, the hospital that developed the model and the hospital that conducted the accuracy test were completely separated.

Many researchers have attempted to determine whether machine-learning models developed for the prediction of one outcome can predict other similar outcomes. For example, some researchers have confirmed that a machine-learning model trained from in-hospital cardiac arrest data can predict unexpected intensive care unit transfer due to deterioration or death without attempted resuscitation.[15,27] We have confirmed that DAMI, which was developed with in-hospital mortality data, can predict 6-month mortality in this study. Because the available data is limited and the outcomes to be predicted are highly diverse, this result is promising to future studies in medical domains and will inspire many researchers.

Several limitations are present in our study. First, deep-learning is known as a “black box.” Although we can fit the deep-learning model by confirming each weight (*w_k_*), we cannot interpret the deep-learning model, in terms of variable importance or the approach to the decision of risk score. Recently, interpretable deep-learning has been studied and will be our next area of study.[46,47] Second, as previously described, deep-learning models rely on the representability of data. One of the most important characteristics of deep-learning is that it uses only the relationship between variables, as opposed to medical knowledge. Because of this, the developed deep-learning-based model can be tied to representativeness of training data and can thus be biased. Hence, it is necessary to validate this model in other environments, we have provided our prediction model as S1 File. Third, deep-learning only uses existing relationships in the data, regardless of whether they are due to causality or not. Fourth, as DAMI cannot be calculated manually, it is more difficult to use than conventional methods such as TIMI and GRACE. However, there are many hospitals using electronic health records (EHR), the DAMI score could be implemented to these EHRs with the S1 File and calculated automatically. Finally, the proportion of STEMI patients in this study was seen to be significantly higher than that of other studies, in which STEMI patients were 30–40%. There could be a risk of bias and overfitting for STEMI. However, the analysis of each group (STEMI and NSTEMI) showed similar patterns of the performance of deep learning model and conventional model for STEMI and NSTEMI.

## Conclusion

In conclusion, we developed and validated a new risk stratification model of AMI based on the deep-learning approach. The DAMI predicted the in-hospital mortality and 12-month mortality of AMI patients more accurately than the existing risk scores and other machine-learning methods. This study showed the feasibility and effectiveness of the deep-learning-based algorithm model for cardiology, which can be a useful tool for precise decision making in daily practice.

## Supporting information

S1 Table Difference in predictor variables between included and excluded study subjects. (DOCX)

S2 Table Importance of variables in derivation data for each algorithm. AUC denotes area under the receiver operating characteristic curve, BMI body mass index, CRP C-reactive protein, CK-MB creatinine kinase-muscle/brain, LDL low density lipoprotein, OHCA out-of-hospital cardiac arrest, and SBP systolic blood pressure.
(DOCX)

S1 File Deep-learning-based risk stratification for the mortality of patients with acute myocardial infarction and materials for validation process. (ZIP)
