# Supplementary material for: Deep-learning-based risk stratification for mortality of patients with acute myocardial infarction
Source: PLoS One. 2019 Oct 31;14(10):e0224502. doi: 10.1371/journal.pone.0224502 (PMC6822714; doi:10.1371/journal.pone.0224502)
Supplement: S1 Table — (DOCX) [file pone.0224502.s001.docx]

**S1 Table.** **Difference in predictor variables between included and excluded study subjects**

|  | **Included subject**  **(N = 22,875)** | **Excluded subject**  **(N = 3,102)** | **P- value** |
| --- | --- | --- | --- |
| Age (mean ± SD) | 63.8 ± 12.7 | 64.0 ± 12.8 | 0.396 |
| Male, number (%) | 16362 (71.5%) | 2206 (71.5%) | 1.000 |
| BMI | 24.0 ± 3.5 | 23.9 ± 3.2 | 0.054 |
| Systolic blood pressure, mmHg | 129.4 ± 27.4 | 129.0 ± 27.2 | 0.435 |
| Heart rate, bpm | 78.3 ± 19.3 | 78.0 ± 19.0 | 0.418 |
| Previous CPR, % | 458 (2.0%) | 56 (1.8%) | 0.521 |
| Killip class at arrival |  |  | 0.175 |
| Class 1 | 17060 (74.6%) | 2346 (74.6%) |  |
| Class 2 | 2934 (12.8%) | 361 (11.8%) |  |
| Class 3 | 1685 (7.4%) | 211 (6.9%) |  |
| Class 4 | 1196 (5.2%) | 152 (5.0%) |  |
| STEMI, % | 12335 (53.9%) | 1680 (54.5%) | 0.540 |
| Glucose, mg/dL | 170.9 ± 81.3 | 171.1 ± 79.6 | 0.941 |
| CK-MB, ng/mL | 117.3 ± 215.5 | 115.8 ± 217.7 | 0.708 |
| Creatinine, mg/dL | 1.16 ± 1.03 | 1.16 ± 1.02 | 0.946 |
| LDL, mg/dL | 114.45 ± 38.4 | 114.3 ±37.6 | 0.856 |
