# Supplementary material for: Deep-learning-based risk stratification for mortality of patients with acute myocardial infarction
Source: PLoS One. 2019 Oct 31;14(10):e0224502. doi: 10.1371/journal.pone.0224502 (PMC6822714; doi:10.1371/journal.pone.0224502)
Supplement: S2 Table — AUC denotes area under the receiver operating characteristic curve, BMI body mass index, CRP C-reactive protein, CK-MB creatinine kinase-muscle/brain, LDL low density lipoprotein, OHCA out-of-hospital cardiac arrest, and SBP systolic blood pressure. (DOCX) [file pone.0224502.s002.docx]

**S2 Table. Importance of variables in derivation data for each algorithm†**

| **Variable Importance** | **Logistic Regression (deviance difference)** | **Random Forest (mean decreased Gini)** | **Deep-Learning (difference in AUC)** |
| --- | --- | --- | --- |
| **1** | Killip class (-19.73) | Age (137.9) | Killip class (1.91) |
| **2** | Age (-4.85) | Glucose (126.4) | SBP (1.24) |
| **3** | OHCA (-3.66) | CK-MB (120.2) | Glucose (1.11) |
| **4** | Creatinine (-3.35) | LDL (115.4) | Heart rate (0.71) |
| **5** | Glucose (-3.09) | Heart rate (111.6) | Creatinine (0.68) |
| **6** | SBP (-2.87) | Creatinine (108.9) | Sex (0.62) |
| **7** | Heart rate (-2.81) | SBP (18.5) | BMI (0.60) |
| **8** | ST elevation (-0.39) | CRP (18.2) | Age (0.52) |
| **9** | CK-MB (-0.21) | BMI (18.1) | CRP (0.49) |
| **10** | LDL (-0.09) | Killip class (18.0) | LDL (0.45) |
| **11** | Sex (-0.06) | OHCA (15.8) | OHCA (0.41) |
| **12** | CRP (-0.03) | ST elevation (15.5) | ST elevation (0.20) |
| **13** | BMI (-0.01) | Sex (2.8) | CK-MB (0.08) |

†AUC denotes area under the receiver operating characteristic curve, BMI body mass index, CRP C-reactive protein, CK-MB creatinine kinase-muscle/brain, LDL low density lipoprotein, OHCA out-of-hospital cardiac arrest, and SBP systolic blood pressure.
