# Supplementary material for: Deep-learning-based risk stratification for mortality of patients with acute myocardial infarction
Source: PLoS One. 2019 Oct 31;14(10):e0224502. doi: 10.1371/journal.pone.0224502 (PMC6822714; doi:10.1371/journal.pone.0224502)
Supplement: S1 File — (ZIP) [file pone.0224502.s003.zip › codingBook.docx]

input_data_x_val_example.csv

| column | variables | data pre-processing | | |  |
| --- | --- | --- | --- | --- | --- |
| 1 | Sex | male | 1 | female | -1 |
| 2 | ST elevation | YES | 1 | NO | 0 |
| 3 | pre CPR | YES | 1 | NO | 0 |
| 4 | Age | normalization for this continous variable | | | |
| 5 | Killip class | normalization for this continous variable | | | |
| 6 | Glucose | normalization for this continous variable | | | |
| 7 | CKMB | normalization for this continous variable | | | |
| 8 | Heart Rate | normalization for this continous variable | | | |
| 9 | Creatinine | normalization for this continous variable | | | |
| 10 | SBP | normalization for this continous variable | | | |
| 11 | CRP | normalization for this continous variable | | | |
| 12 | LDL | normalization for this continous variable | | | |
| 13 | BMI | normalization for this continous variable | | | |
| output_data_y_val_example.csv | | | | | |
| In-hospital mortality | Yes | 1 | No | 0 |  |
