# Supplementary figures and images for: Deep-learning-based risk stratification for mortality of patients with acute myocardial infarction
Source: PLoS One. 2019 Oct 31;14(10):e0224502. doi: 10.1371/journal.pone.0224502 (PMC6822714; doi:10.1371/journal.pone.0224502)

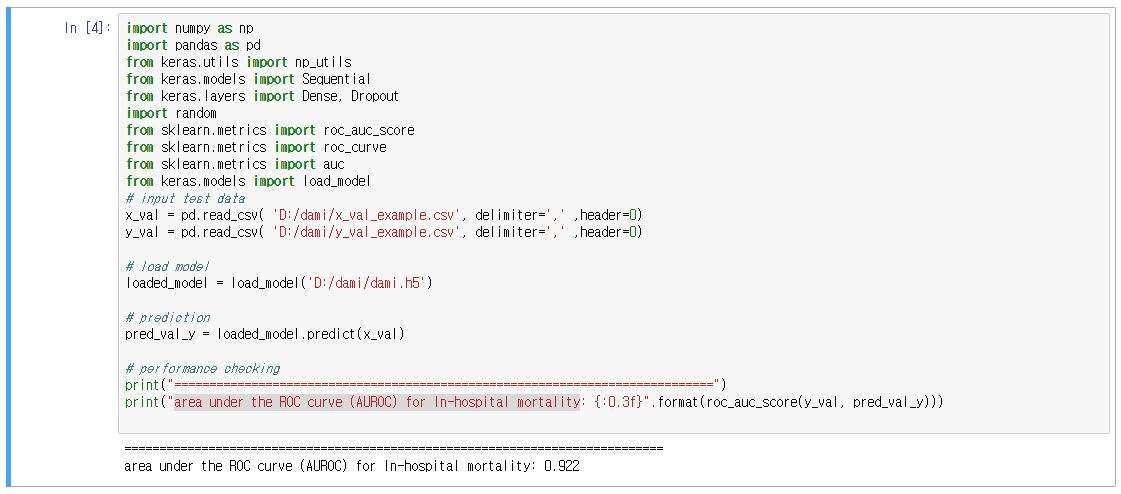

Supplement: S1 File — (ZIP) [file pone.0224502.s003.zip › example_image_of_validation.JPG]
